# Moderate Effects of School-Based Time Increasing Physical Education Intervention on Physical Fitness and Activity of 7-Year Pupils—A Report from a Follow-Up of a HCSC Study

**DOI:** 10.3390/children9060882

**Published:** 2022-06-13

**Authors:** Paweł Lisowski, Adam Kantanista, Michał Bronikowski

**Affiliations:** 1 Department of School Practice, Poznań University of Physical Education, 61-871 Poznań, Poland; 2 Department of Physical Education and Lifelong Sports, Poznań University of Physical Education, 61-871 Poznań, Poland; kantanista@awf.poznan.pl; 3 Department of Didactics of Physical Activity, Poznań University of Physical Education, 61-871 Poznań, Poland; bronikowski@awf.poznan.pl

**Keywords:** children, physical fitness, physical activity, children, school intervention

## Abstract

We evaluated the effectiveness of a 15-week intervention that increased from three to five lessons of physical education (PE) a week on 7-year-old boys’ and girls’ physical fitness (PF), physical activity (PA) and sedentary behaviour on week and weekend days. A total of 212 first grade pupils (mean age 6.95 ± 0.43) from two urban schools in Poznań were randomly assigned to the experimental or control groups. The PF was measured with a battery of field tests, while health-related behaviours were assessed with the Healthy Children in Sound Communities questionnaire. There were some interaction effects noticed in the PF scores in the case of a 20-min run for boys (F_2,196_ = 5.29, *p* = 0.0058) and for girls (F_2,220_ = 3.31, *p* = 0.0382) and the sit-ups test for boys (F_2,196_ = 1.93, *p* = 0.1478) and for girls (F_2,220_ = 3.98, *p* = 0.0201) and for the sit and reach test in the case of girls (F_2,220_ = 3.98, *p* = 0.0201). In terms of outdoor PA levels, there were no major differences between any of the examined groups. Differences were found between girls from the experimental and control groups in the post-test (*p* = 0.0107) and follow-up (*p* = 0.0390) during the weekdays, with no differences between the groups of boys. Despite the moderate effects of the extended PE time programme right after the intervention, there were some indications of progress in the follow-up experiments.

## 1. Introduction

Decreasing the PA levels of successive generations of schoolchildren has become a major concern and threat in modern societies heavily overwhelmed by the use of digital technology and sedentary behaviours [1]. Failing to meet the World Health Organization’s [2] recommended daily doses—at least 60 min of moderate-to-vigorous PA (MVPA) in adolescents and children or 300 min per week generally for everyone who can—may result in the long-term development of noncommunicable diseases like hypertension, coronary heart disease, stroke, diabetes, cancer, or depression [3]. The proportion of children and adolescents meeting the recommended MVPA doses is unacceptably low. In global documents on that issue, it is suggested that school should play a more important role in the health and well-being of school pupils. Every school should be a health-promoting school, using a whole-school system [4], since homes and schools remain ideal settings in which to modify unhealthy eating habits and promote PA, specifically by increasing the healthy nutrition standards and school-based PA time targeting at-risk groups.

The decline of MVPA observed since 2014 in findings from the recent international Health Behavior in School-Aged Children (HBSC) study is deeply worrying [5]. Another recent global report on children PA [6] provides additional evidence that the situation regarding PA in children is very concerning. Unless a major shift to a more active lifestyle happens soon, a high rate of noncommunicable diseases can be anticipated when this generation of children reaches adulthood. Among the most disturbing factors are the lack of free active play and, at the same time, limited opportunities to participate in organized PA, specifically in school settings, with increasing screentime adding to a generally high amount of sedentary behaviours. An analysis on the cross-sectional longitudinal studies on PA in youths indicated that it is organized sports and active travel that contribute more to the daily MVPA than PE [7].

Some can see the problem why PA levels in youth have been decreasing recently, even more rapidly due to the extensive use of new tools of technology, which also has the effect of increased social interaction time online [1]. A lowered PA decreases the level of PF and (dis)enables people to be physically active, providing they have the range of appropriate motor skills required for particular activities/sports [8].

This should be taken into account while programming a plan of activities for kindergarten and early age school children, as the participation of children in PE contributes to a significantly higher duration of MVPA during the school day. However, it is not known whether this increase in MVPA is at the expense of light physical activity (LPA) or sedentary behaviours [9]. What is known from the research is that there is no significant association between LPA and fundamental motor skills and total locomotor skills, but there is evidence of associations between certain motor skills’ competency and the intensity of PA [10]. In a study [11] on Polish 5-year-old children, a higher level of gross motor skills was noticed in girls. This was influenced mainly by their locomotor skills, but no significant differences in their locomotor skills were noted when analysed by different levels of PF.

On the other hand, in a review paper on the effectiveness of school-based interventions [8], we learned that the positive developments of some components of PF were observed where the primary objective of the intervention was to reach and maintain health-related PF focused on factors such as: the BMI, cardiorespiratory endurance (aerobic endurance), muscular strength, muscular endurance, and flexibility. For example, the study of Xu et al. [12] showed that the level of flexibility of normal weight adolescents was worse compared to their obese peers. Casonatto et al. [13] found that abdominal obesity might affect the lower back and hamstring flexibility and hamper the trunk from the extreme reach position. The study of Tokmatidis [14] found that a higher BMI was strongly associated with the quality of the performances in the fitness tests, except for flexibility. The link between a lowered PF and PA is clear. One of the mediating factors responsible for these inactivity-related problems in youths is extensive screentime and the role played by the media and the Internet.

In Finland, in the 1970s, the total TV viewing time for adolescents was, on average, 1.30 h/day, while, for example, in the past decade, their screentime has been estimated to be as high as 5 to 6 h/day. [15]. In Poland, a study on a big sample of Polish children aged 6 to 7 years by Ciesla et al. [16] indicated a statistical significance (although low) between the BMI and PF, which negatively affected the levels of the health-related components. A similar negative effect was observed in the case of computer game times on flexibility, explosive power, and trunk muscle strength. The authors concluded that the observed decrease of fitness when combined with other external factors (inactive forms of spending leisure time/PA) may be responsible for the negative drop of their fitness levels, and its side effect is connected with an excessive body weight and allows for growth of the prevalence of overweight-related problems. This situation is not just country-specific [17]. The implications are numerous.

Excessive body weight implies lower PF levels, which have been declining for decades in children [18]. Furthermore, there is evidence that the prevalence of other health-related fitness attributes, such as cardiorespiratory endurance, is suboptimal and has been declining since the 1970s [19,20]. There is also a relationship between motor skills and PF from a long-time perspective. The study by Kemper et al. [21] showed that the motor skills level in adolescents is related to adult PF only in a group with maximal aerobic power. In children, only pupils with lower PF levels improved statistically significantly in cardiorespiratory fitness [22]. The study by Holfelder and Schott [23] provided evidence based on cross-sectional studies of a positive relationship between fundamental motor skills and organized PA, whereas the research by Barnett et al. [24] revealed the cause–effect relationship of perceived sport competences and motor skills proficiency with the level of PA and fitness in children and adolescents. The results also showed that a low motor competency turns children off from a potential interest in any form of PA, while a positively perceived motor competency in 8 year olds provided long-term motivation for PA [25,26] and thus helped to maintain a reasonably higher level of PF for longer.

Preventing a further decline in PA of children and youths has become one of the major objectives of the WHO [2]. In many countries, public health policies focus on the development of preventing programmes involving enhanced health education via the school system (PE and health curricula changes) and/or different intervention programmes. They differ in terms of duration (from 2-week actions to regular whole-year programmes) and contents (increased PE time or/and health education classes and nutrition training) and also in modes of delivering (school-based, active breaks, extracurricular activities, fitness centres, or active commuting to and from school) involving children and, sometimes, the whole families. The research findings on the effectiveness of various solutions are sparse and inconclusive [8,27,28].

The school setting is a reasonably easily accessible platform for introducing any form of curricular or interventional changes aimed at enhancing the health-related awareness of a potentially broad cohort of children and youths. Modifications of PE lesson contents seem to be the easiest way—having professionals on their side, health policy planners can design a programme tailored to the needs of particular age groups. However, the effectiveness of such solutions depends heavily on the engagement of PE teachers. Barrett et al. [29] measured the impact of the national active PE policy in the USA (at least 50% of lesson time spent on MVPA) on the PA, BMI, and healthcare costs in a cohort of school children aged 6–11 years and found that such policy could have an impact (although small) on the PA levels in the population and potentially lead to a lower BMI and reduce health system expenditures over a 10-year period. In another analysis, reviewing thirty pooled and objectively measured PA experiments on children, Metcalf et al. [30] found that such interventions have only a small effect (an increase of 4 min of walking/running extra time added per day, on average) on the children’s overall PA levels, which may explain the limited success in reducing their BMI or increasing their PF. Others who tried to enhance the overall sound development through gross motor skills [31,32,33] proved that school-based interventions can lead to an increase in PA, PF, and the prevention of obesity. However, there are also studies [34,35,36] that have reported a low or null effect of PA interventions on children in variables such as fitness, the PA levels, and sedentary behaviours, especially when delivering PE of quality was an issue.

It is important to report some evidence-based outcomes to indicate the (in)effectiveness of a multicomponent intervention within an integrated approach to the physical and health education school process. Therefore, we designed a study that aimed to explore and evaluate the effectiveness of 15 weeks of increased PE time intervention on 7-year-old boys’ and girls’ PF, PA, and time spent on sedentary behaviour on week and weekend days, including the long-term effects 12 months after cessation of the intervention programme. We also wanted to compare own results with the ones from similar studies on cohorts of children from Germany and the Netherlands [37].

## 2. Materials and Methods

The study is a part of the European Union project (EAC/21/2009/033—Healthy Children in Sound Communities—HCSC) carried out in 6 European countries (Germany, the Netherlands, Poland, Czech, Italy, and England) between the years 2010 and 2012, which was coordinated by the Willibald Gebhardt Research Institute (Münster, Germany). The idea of HCSC was based on a joint effort of a community network of stakeholders, including local educational authorities, sports clubs, families, and appropriate departments of municipality, to all provide health education both through curricular and extracurricular activities in the school PE setting. The details of the project have already been described elsewhere [32].

### 2.1. Participants

In Poland, the study included data collected from 212 first grade pupils (mean age 6.95 ± 0.43; body mass 25.4 ± 4.86; body height 124.8 ± 5.88). Six classes (*n* = 151) were randomly assigned to the intervention group, and three classes (*n* = 61) formed a control group. The participants were recruited from two preselected urban schools of the Poznań municipality by contacting the school headmasters and then the parents and guardians of the pupils, who were given explanations and informed about the purpose of the programme and about the anonymous and voluntary nature of their children’s participation. There was a 96% acceptance and response rate. Written consent was obtained. The study was approved by the Bioethical Committee of the University of Medical Sciences in Poznań (decision No. 552/11) and was performed in accordance with the Declaration of Helsinki for data collection and custody protection.

### 2.2. Research Tools

Anthropometric and PF measures were performed during the week before the programme started, right after the 15-week programme ended, and once again after a full year of cessation of the programme.

Anthropometric measures (body height and weight) were collected during PE classes and recorded by trained research assistants at each of the examination terms. Body height was measured to the nearest 0.5 cm (cm) using a stadiometer, and body weight was measured to the nearest 0.1 kg (kg) using electronic scales (Tanita Corporation, Tokyo, Japan), with the participant wearing minimal clothing.

PF was measured during one PE class, after a regular 5–7-min warm-up, with the use of the five field tests: (1) strength endurance was measured by the number of sit-ups in a time of 40 s. The purpose of the test was to measure the endurance of the abdominal and hip-flexor muscles. It was performed on a gymnastic mat with knees bent at right angles, and with feet resting flat on the floor, hooked underneath a gym ladder. The fingers were interlocked behind the head. The number of correctly performed sit-ups in 40 s was recorded; (2) cardiorespiratory fitness was measured by a 6-min run/walk around a rectangle with 9 × 18-m dimensions and the distance covered in metres was registered for each pupil; (3) standing broad jump tests their explosive power. A pupil performed a jump from standing with both feet on the floor, and from behind the line, he jumped for the longest distance in cm; (4) agility (including a speed component) was evaluated by a 20-m run as fast as possible on the signal between two lines 20 m apart; and (5) the sit and reach test assessed the flexibility of the lower back and hamstring muscles. The test involved sitting on the floor with legs stretched out in front and feet placed flat against a box, shoulder-width apart. Pupils were asked to reach forward as far as possible, along with a measuring tape. Except for the explosive power assessment (where the better result of two tests was taken), all other tests were recorded once. The whole procedure was described earlier in detail [37].

The assessment of a healthy lifestyle took place in the quiet environment of a classroom setting with the use of a questionnaire, “Healthy Children in Sound Communities”, which was completed by the parents/guardians of the children participating in the study and took approximately 15 min. Before the programme started, parents were asked to fill in the questionnaire (September 2011). They were also asked to answer the same questions after the 15-week programme ended (January 2012) and once again after a full year of cessation of the programme (January 2013). They had to answer the following question: How often does your child play outdoors (outside school)? With the range of frequency provided for selection from 1 = less than once a week, 2 = 1 to 2 times a week, 3 = 3 to 4 times a week, 4 = 5 to 6 times a week, and 5 = every day. Another question concerned sedentary behaviour: How long does your child play computer games/game console games each day? Separately for weekdays and weekends, we analysed the answers with the time duration scale provided for the selections ranging from 5 = less than half an hour, 4 = between half and one hour, 3 = between one and two hours, 2 = between two and three hours, and 1 = three or more hours a day.

### 2.3. Study Design and Intervention Procedure

An experimental intervention with parallel experimental and control groups was designed. In the intervention, the three 45-min lessons of PE a week schedule (as in the PE school curricula led by an elementary teacher) was extended with two additional 45-min lessons of physical activities in the form of a regular PE lesson. These lessons were included in the normal school timetable and were led by a local sports club staff to balance fitness deficits and encourage the development of individual PA and health-related patterns of behaviour (with an intensity similar to the ones in the regular PE classes led by a trained specialist). The idea of including the staff of local clubs was the main purpose of the Healthy Children in Sound Communities project, which intended to bring local health-related stakeholders to the school setting. The rationale behind this was the lack of proper PE-trained specialists working with children in elementary education, where all the classes are usually led by a general elementary education teacher.

Therefore, pupils were provided with a 45-min PE lesson five times a week, literally on every school day. Only pupils from the experimental groups (without parents) participated in the intervention programme. The intervention was carried out over15 consecutive weeks in the school year 2011/2012, from September to January. The follow-up examination took place 12 months after the cessations of the intervention programme in January 2013. The control group had three 45-min PE lessons scheduled a week due to the PE school curricula led by a general elementary education teacher.

### 2.4. Statistical Analysis

To assess the changes of fitness in the examined groups in relation to the examination terms, the analysis of variance (ANOVA) test was used with Tukey’s post hoc test. To compare the results of the questionnaire items in three examination terms, Friedman’s ANOVA test was used with post-hoc analysis between the terms. For testing the differences between the medians in the groups of boys and separately between the groups of girls in all three terms, the Mann–Whitney *U* test was used for the questionnaire items. The statistical significance was set at *p* < 0.05. Analyses were carried out with the use of STATISTICA software, version 13 (StatSoft, Krakow, Poland).

## 3. Results

The PF was measured during three different terms: pre-test, post-test, and follow-up (Table 1).

For the 20-m run, in boys, there were no statistically significant differences (*p* ≥ 0.05) between the experimental and control groups. There were statistically significant differences between the terms of examination (F_2,196_ = 19.92, *p* < 0.0001), statistically significant differences between the pre-test and follow-up (*p* < 0.0001), and statistically significant differences between the post-test and follow-up (*p* < 0.0001). There was also an interaction effect on the boys (F_2,196_ = 5.29, *p* = 0.0058); statistically significant differences were between pre-test and follow-up (*p* < 0.0001) and between the post-test and follow-up (*p* < 0.0001) in the experimental group. For the 20-m run, in girls, there were no statistically significant differences (*p* ≥ 0.05) between the experimental and control groups. There were statistically significant differences between the terms of examination (F_2,220_ = 19.86, *p* < 0.0001), statistically significant differences noted between the pre-test and follow-up (*p* < 0.0001), and statistically significant differences between the post-test and follow-up (*p* < 0.0001). There was also an interaction effect on the girls (F_2,220_ = 3.31, *p* = 0.0382); statistically significant differences were noted between the pre-test and follow-up (*p* < 0.0001) and between the post-test and follow-up (*p* < 0.0001) in the experimental group.

For the 6-min run, in boys, there were statistically significant differences (F_1,98_ = 7.25, *p* = 0.0084) between the experimental and control groups. There were statistically significant differences between the terms of examination (F_2,196_ = 10.19, *p* = 0.0001), statistically significant differences noted between the pre-test and post-test (*p* = 0.0001), and statistically significant differences between the post-test and follow-up (*p* < 0.0001). There was no interaction effect on the boys (F_2,196_ = 2.20, *p* = 0.1132). For the 6-min run, in girls, there were no statistically significant differences (*p* ≥ 0.05) between the experimental and control groups. There were statistically significant differences between the terms of examination (F_2,220_ = 16.16, *p* < 0.0001), statistically significant differences noted between the pre-test and post-test (*p* < 0.0001), and statistically significant differences between the post-test and follow-up (*p* < 0.0001). There was no interaction effect on the girls (F_2,220_ = 0.33, *p* = 0.7178).

For standing broad jump, in boys, there were no statistically significant differences (*p* ≥ 0.05) between the experimental and control groups. There were statistically significant differences between the terms of examination (F_2,196_ = 17.97, *p* < 0.0001), statistically significant differences noted between the pre-test and follow-up (*p* < 0.0001), and statistically significant differences between the post-test and follow-up (*p* < 0.0001). There was no interaction effect on the boys (F_2,196_ = 0.14, *p* = 0.8734). For standing broad jump, in girls, there were no statistically significant differences (*p* ≥ 0.05) between the experimental and control groups. There were statistically significant differences between the terms of examination (F_2,220_ = 4.25, *p* = 0.0154) and statistically significant differences noted between the post-test and follow-up (*p* = 0.0043). There was no interaction effect on the girls (F_2,220_ = 0.05, *p* = 0.9548).

For sit-ups, in boys, there were statistically significant differences (F_1,98_ = 7.03, *p* = 0.0094) between the experimental and control groups. There were statistically significant differences between the terms of examination (F_2,196_ = 40.03, *p* < 0.0001), statistically significant differences noted between the pre-test and post-test (*p* < 0.0001), statistically significant differences between the pre-test and follow-up (*p* < 0.0001), and statistically significant differences between the post-test and follow-up (*p* = 0.0040). There was also an interaction effect on the boys (F_2,196_ = 3.96, *p* = 0.0206), statistically significant differences between the pre-test and post-test (*p* < 0.0001), statistically significant differences between the pre-test and follow-up (*p* < 0.0001), and statistically significant differences between the post-test and follow-up (*p* = 0.0275) in the experimental group and, also, statistically significant differences between the pre-test and follow-up (*p* = 0.0048) in the control group. For sit-ups, in girls, there were no statistically significant differences (*p* ≥ 0.05) between the experimental and control groups. There were statistically significant differences between the terms of examination (F_2,220_ = 68.01, *p* < 0.0001), statistically significant differences noted between the pre-test and post-test (*p* < 0.0001), statistically significant differences between the pre-test and follow-up (*p* < 0.0001), and statistically significant differences between the post-test and follow-up (*p* < 0.0001). There was also an interaction effect on the girls (F_2,220_ = 3.09, *p* = 0.0475), with statistically significant differences between the pre-test and post-test (*p* < 0.0001), statistically significant differences between the pre-test and follow-up (*p* < 0.0001), and statistically significant differences between the post-test and follow-up (*p* < 0.0001) in the experimental group and, also, between the pre-test and post-test (*p* = 0.0017) and between the pre-test and follow-up (*p* < 0.0001) in the control group.

For sit-and-reach, in boys, there were no statistically significant differences (*p* ≥ 0.05) between the experimental and control groups. There were statistically significant differences between the terms of examination (F_2,196_ = 16.42, *p* < 0.0001), statistically significant differences noted between the pre-test and follow-up (*p* < 0.0001), and statistically significant differences between the post-test and follow-up (*p* < 0.0001). There was no interaction effect on the boys (F_2,196_ = 1.93, *p* = 0.1478). For sit-and-reach, in girls, there were no statistically significant differences (*p* ≥ 0.05) between the experimental and control groups. There were statistically significant differences between the terms of examination (F_2,220_ = 9.03, *p* = 0.0002) and statistically significant differences noted between the post-test and follow-up (*p* = 0.0007). There was an interaction effect on the girls (F_2,220_ = 3.98, *p* = 0.0201); statistically significant differences were between the pre-test and follow-up (*p* = 0.0054) and between the post-test and follow-up (*p* = 0.0099) in the control group.

In the case of the frequency of outdoor PA among the examined children (Table 2), it was observed that, in both groups, experimental and control, there were some statistically significant differences between the terms (pre-test, post-test, and follow-up), indicated by ANOVA testing, but the further analysis with the post-hoc test did not show any significance between the examination terms. Moreover, there were no statistically significant differences between the experimental and control groups in boys and in girls in either of the examined terms.

The amounts of screentime on weekdays and weekend days are presented in Table 3. In the case of weekdays, ANOVA indicated some differences in the experimental boys (*p* = 0.0393), experimental girls (*p* = 0.0465), and control girls (*p* = 0.0460), but a further in-depth analysis with the post-hoc test did not show any statistically significant differences between the examined terms in any of the cases. During weekdays, differences were found between girls from the experimental and control groups in the post-test (*p* = 0.0107) and follow-up (*p* = 0.0390). While there were no such differences observed in the boys. During weekend days there, were no statistically significant differences between any of the terms. There were also no statistically valuable differences within the genders (within the experimental and control boys and within the experimental and control girls) in any of the examined terms.

## 4. Discussion

Although school-based interventions are believed to be the most suitable ways of increasing low PA and, thus, lifting the fitness levels in school-aged children, despite their universal applicability, it is difficult to find an optimal strategy.

In our study, in the first term of the examination (pre-test), there were no major statistically significant differences between the examined groups of boys (except for the 6-min run and sit-ups, both in favour of the experimental boys). No such differences were found between the groups of girls. However, some changes were noticed right after the end of the intervention programme (post-test) and in the follow-up examination. In the boys, there was a statistically significant difference observed in the 20-m run between the terms of examination, and an interaction effect was noted, with a significant improvement in follow-up in the experimental group. In girls, an interaction effect was also observed with a drop in the post-test but with significant improvement in the follow-up testing. In the 6-min run, a drop in the scores in the post-test was noticed in all the groups of boys and girls. For the sit-ups test, there was an interaction effect indicated in both groups of boys (experimental and control), scoring better in the post-test and even better in the follow-up. An interaction effect in the girls showed a similar pattern of scoring in both groups over the terms of examination, with the experimental girls again being better during both the post- and follow-up terms. In the case of the sit-and-reach test, there was an interaction effect noticed in the girls, where the experimental group of girls had better results across the examination terms than the control girls, who noted a significant drop along the examination terms.

Some improvements in the level of fitness were noticed (statistically significant in speed), especially in the follow-up examination in the experimental groups, but also, some increasing trends (such as in the case of endurance) were observed. It is hard to say whether it was a direct effect of the programme, considering that, right after the programme, some of the scores were lower even than the ones in the beginning. However, worth mentioning is the fact that a similar situation was observed in the control group, where the lowered levels of the scores by boys and girls also stayed equally low during the follow-up testing, which may indicate that this was the normal rate of development of these fitness components in children of this age. In that case, the increase in the experimental group both in boys and girls could be accredited to a long-term awareness awakened by the intervention programme and some extra added physically active leisure time. Additionally, trunk muscles (sit-ups test) differentiated between the groups of boys and girls in the post-test and follow-up in favour of the experimental ones, which can be considered as both a direct and a long-term effect of the programme.

It is worth looking at cohorts of children (similar ages) from German and the Netherlands [37] who have achieved comparable results to the Polish children.

Between German and Dutch children, it was the German ones who gained significantly better results in the 20-m run, whereas Dutch children scored significantly better on the sit-ups (trunk muscles strength) and sit-and-reach (flexibility) tests. In both cohorts (German and Dutch), the significant increase after the intervention (the same program: 15 weeks of five times a week 45-min PE) was noticed in the case of endurance, coordination, velocity, and force tasks. In the case of endurance (6-min run), the German pupils increased the scores from 876 to 944 m in distance, and the Dutch ones from 873 to 914 m in distance, and in both cases, it was statistically significant, whereas the Polish pupils from the experimental groups dropped from 844 to 811 m in boys and 772 to 739 m in distance in girls, but the changes were not statically significant. A similar drop was observed in both control groups (boys and girls).

Therefore, we can assume that the intervention programme content was not stimulating enough to cause any reaction in terms of endurance in a short time and that the scores gained by the Polish cohort were due to the normal rate of growth of pupils at that age. Another possible explanation may be found in the age differences between the cohorts (in Poland, the mean age was below 7 and just above 7 years old at the end of the 15-week intervention program, while the mean age of the German and Dutch pupils was above 7 during the first testing and around 8 during the second testing). What is worth noticing is the fact that Polish pupils improved their endurance scores one year after the cessation of the intervention by increasing to a 857-m distance (experimental boys) and 788 m for girls from the experimental group, while, in the control groups, it was only 773 m for the boys and 753 m for the girls. German and Dutch pupils also scored better than Polish pupils in the 20-m run (agility), but Polish pupils scored better in sit-ups (trunk muscles strength). In the sit-and-reach (flexibility) test, the scores between the cohorts from these three countries were comparable.

Some studies [38] have reported interventions that did not succeed in increasing the PA levels or in other selected factors (such as, e.g., reducing body fat or PF components), and the authors suggested this could be due to the poor delivery of the intervention contents or/and insufficient intensity of the activities. In another paper, the authors [31] reviewed a number of school-based interventions to report some positive effects on in-school and out-of-school, as well as overall, PA, but this was more likely with older children and youths and with an increase in fitness levels only in 6 out of 11 interventions, which was accredited to measurement technique differences. Interventions in which trials assessing aerobic fitness have been carried out with the use of VO_2_ max methods have been more effective than the ones which used field tests. This could also refer to our project.

Eather et al. [39], in their report from a randomized eight weeks of Fit-4-Fun intervention in primary school children, suggested positive effects in PF components (cardiorespiratory fitness, flexibility, and muscular strength); body composition; and the levels of PA, and the differences in the control group remained at the 6-month follow-up examination. According to the authors, this positive (and lasting) effect was achieved due to the use of a multi-component school-based approach with curriculum, environment, and family support. This could also be the case with our intervention in the future if parents become active agents in the programme.

Concerning our results of the time spent playing outdoors, the findings indicate some differences between the terms of examination in the experimental group, both in boys and girls. However, the post-hoc analysis did not reveal any statistical significance. Therefore, we can practically conclude that the time spent playing outdoors did not change significantly as an effect of the intervention programme. Generally, boys and girls aged 7 from both groups spent time playing outdoors three to four times a week, on average, which may be an indication of their natural need for playing with their peers in their leisure time. The median for sedentary behaviour on weekdays in the experimental boys was lower during both the post-test and follow-up test. We can say there was also no clear and strong direct effect (the one right after the intervention programme) on reducing the amount of sedentary behaviours in the experimental group. It is worth noticing, however, that the median in boys went up from less than half an hour to one to two hours a week. This amount of time spent was also similar on weekend days. Boys from the control group also spent the same time on playing computer/console games both on week and weekend days. A comparable situation was observed in girls, which might be typical for children of this age.

Although we did not include into the analysis the role of the parents, due to the lack of full data available in all three examination terms, other studies assessing the effectiveness of interventions targeted at improving PA and reducing screen times in children have shown how important the role is played by the parents. This factor should be included in further studies. For example, Sigmundova et al. [9] found that, in families where both parents met the WHO PA weekly recommendations, the children were five times more likely (in relation to their fathers) and three times more likely (in relations to their mothers) to meet the WHO PA weekly age-specific recommendations themselves, as compared to the children of less active parents. Children whose parents introduced restrictions on TV time watched it 2 h less than their peers, whereas the risk increased with low-restriction families [40]. The risk also grew with the increase in TV viewing time where parents viewing was concerned.

The present paper describes the outcomes of a school-based intervention programme (increased from three to five times a week PE classes in first grade children). There were some positive changes noticed, but the lack of extensive progress in PF of the 7-years-old boys and girls who participated in the presented intervention may be attributed to a few aspects. First, it is possible that the 15-week duration time of the intervention was not sufficient to cause permanent changes in the fitness levels. It is also possible that the contents of the intervention programme might have been an issue. From other studies [14], it is clear that cardiorespiratory fitness or some other components such as muscular strength or flexibility may be increased by focusing the intervention programme contents on those aspects and applying the rightful doses of exercising. However, in our case, the activities and tasks used in the programme were mainly based on playing and movement games specific to the age and educational objectives of the PE curricula of first grade pupils. This has probably brought out more creativity and a fun context during the classes but was not sufficient stimuli to elevate the levels of fitness in the examined components. A more spectacular increase in fitness (at least during the post-test examination) could have probably been achieved easier if the programme included an exercise-like training scheme, but that was not the first aim of the intervention. We opted for a more fun and emotional engagement, hoping for lasting effects, especially in terms of the PA. Hopefully, higher scores in the endurance test in the follow-up in both experimental boys and girls could be accredited to the higher frequency of undertaking PA by this group based on the fun and enjoyment they experienced during the intervention programme. As such, this can be seen as a long-term outcome of the intervention. Unfortunately, we did not measure any emotional or psychosocial factors in our study, so it is impossible to determine any changes in those aspects, but we believe that this should be included in further research and intervention studies, especially on young children.

With a sedentary screentime, the findings of our study did not show any direct or long-term significant changes of behaviours, but one needs to remember that there might have been some factors interfering (educational setting, parenting style and restriction, access to modern technology, etc.). An analysis of qualitative studies by Tremblay et al. [41] indicated that a dose–response relation between increased sedentary behaviour and unfavourable health outcomes, such as watching TV for more than 2 h per day, were associated with an unhealthy body composition, decreased fitness, lowered self-esteem, or prosocial behaviour, with decreased academic achievements. On the other hand, there is evidence that decreasing any type of sedentary time is associated with a lower health risk in children and youths, especially that lowering the sedentary time leads to reductions in many health-related negative outcomes, like the BMI, distribution of body fat, blood pressure, and psychological ones like depression [42,43].

A huge role in combat against unhealthy sedentary behaviours is played by modern technology. Over the past 25 years, the changes in this area have been rapid, allowing no time to adjust either the educational or parenting styles of dealing with it. This makes it probably the main reason for the growth of more sedentary lifestyle behaviours found among youths that, in turn, may have an effect on the PF [22]. On the one hand, the accessibility of multiple forms of screen-based technology (hand-held and portable devices) increases the opportunity to play, watch, and listen at the same time. However, it is unknown yet how this multi-screen multi-task combined time will impact children’s health, both in mental and physical terms. At the same time, some of these new technologies are viewed as opportunities for increasing the PA time [44], which effects the rapid increase in gaming market devices and e-activities such as: exergaming (active video games), social media, mobile device apps, health wearables, mobile games, augmented reality games, global positioning and geographic information systems (GPS/GIS), and virtual reality [45]. However, long-term psychological or social side effects of such forms of activity (indoor, alone, and in front of a screen) are yet to be determined.

Although it is an experimental intervention study, some limitations of the study have to be acknowledged. The relatively small sample sizes of the groups might be one concern. Additionally, children at the age of 7 are still in the process of biological and psychophysical development, and thus, many motor-related aspects are fluctuating with their individual biological rate of maturation, so some fitness scores should be looked at as being potentially influenced by other means. Similarly, the answers to the questions on leisure time activities of children asked to parents should also be treated with caution, as parents tend to underrate their children’s activities. In the study, we probably could have used other research instruments, such as, e.g., a PA screening measure test, which is easy to implement in a school setting [46], and confronted it with an objectively measured PA collected by activity monitors. This could give more accurate estimates of the activity times during week and weekend days, but we had to use the same research methodology and tools as in the other HCSC studies. On the other hand, in report papers from various other PA interventions, it was usually positive effects that were published, while studies reporting moderate or neutral effects were not published so often. For that reason, this can also be considered as one of the strengths of this report.

In the future, further studies should be aimed at differencing the relation between the frequency, duration, and intensity of PA sessions/lessons and also examining the effects of the family context on the structure of the weekly overall PA and sedentary time and related behaviours.

## 5. Conclusions

This study shows that there were no major effects of a 15-week intervention of an extended five times a week PE programme in terms of physical fitness, leisure time PA, or sedentary behaviours right after delivering them. However, there were some interaction effects noticed, specifically during the follow-up examination (speed and trunk muscles). Future studies with a larger sample size should examine whether interventions with different durations and varied contents may have different effects on PA and sedentary behaviours. The emotional and psychosocial factors (possibly also including parents) should also be included in further research and intervention studies, especially at the moment of transition from kindergarten to school.

## Figures and Tables

**Table 1:** Differences between the experimental and control groups in the fitness test (5 items).

		Boys		Girls	
		Experimental	Control	Experimental	Control
20-m run (s) ^b,c^	Pre-test	4.89 ± 0.42	4.80 ± 0.44	5.05 ± 0.41	4.97 ± 0.42
	Post-test	4.92 ± 0.48	4.95 ± 0.31	5.13 ± 0.42	5.03 ± 0.36
	Follow-up	4.64 ± 0.44 ^d,e^	4.78 ± 0.35	4.82 ± 0.47 ^d,e^	4.89 ± 0.38
6-min run (m) ^a,b,c^	Pre-test	844.60 ± 115.56	793.24 ± 111.89	772.54 ± 105.27	750.94 ± 117.48
	Post-test	811.52 ± 121.94	756.00 ± 101.02	739.13 ± 118.77	710.44 ± 96.36
	Follow-up	857.54 ± 120.96	773.13 ± 112.28	788.85 ± 124.61	753.22 ± 94.68
Standing broad jump (m) ^b,c^	Pre-test	1.07 ± 0.18	1.06 ± 0.18	1.01 ± 0.20	0.96 ± 0.21
	Post-test	1.07 ± 0.18	1.05 ± 0.15	0.99 ± 0.18	0.94 ± 0.15
	Follow-up	1.16 ± 0.18	1.13 ± 0.19	1.04 ± 0.22	0.99 ± 0.16
Sit-ups (number) ^a,b,c^	Pre-test	17.20 ± 4.56	15.97 ± 5.12	15.30 ± 4.72	15.41 ± 5.15
	Post-test	20.51 ± 5.33 ^d^	17.62 ± 3.61	18.76 ± 4.84 ^d^	17.97 ± 3.62 ^d^
	Follow-up	21.85 ± 5.18 ^d,e^	18.41 ± 3.64 ^d^	20.79 ± 5.41 ^d,e^	18.94 ± 3.35 ^d^
Sit-and-reach (cm) ^b,c^	Pre-test	14.29 ± 5.35	14.45 ± 4.36	17.05 ± 5.64	16.63 ± 3.74
	Post-test	14.65 ± 6.09	13.52 ± 4.44	17.69 ± 5.82	16.53 ± 3.78
	Follow-up	12.91 ± 5.20	12.31 ± 4.11	16.93 ± 6.24	14.69 ± 4.13 ^d,e^

^a^ Statistically significant between the experimental and control groups (boys), ^b^ statistically significant between the terms of examination (boys), ^c^ statistically significant between the terms of examination (girls), ^d^ significantly different from the pre-test, and ^e^ significantly different from the post-test.

**Table 2:** Comparison of the values (median) of outdoor activity with Friedman’s ANOVA and within the genders (with Mann–Whitney *U*) in the examined 7-year-old children.

	Group	Pre-Test Me	Post-Test Me	Follow-Up Me	p_1_
Boys	Experimental	3.0	4.0	3.0	0.0291
	Control	3.0	3.0	3.0	ns
Differences between groups	p_2_	ns	ns	Ns	
Girls	Experimental	3.0	3.0	3.0	ns
	Control	3.0	3.0	3.0	ns
Differences between groups	p_2_	ns	ns	Ns	

ns—not statistically significant, p_1_ = Friedman’s ANOVA, and p_2_ = Mann–Whitney *U*.

**Table 3:** Comparison of the values (median) of the screentime with Friedman’s ANOVA and within the genders (with Mann–Whitney *U*) in the examined 7-year-old children.

	Group	Pre-Test Me	Post-Test Me	Follow-Up Me	p_1_
Weekdays					
Boys	Experimental	5.0	4.5	4.0	0.0393
	Control	4.0	4.0	4.0	ns
Differences between groups	p_2_	ns	ns	ns	
Girls	Experimental	5.0	5.0	4.0	0.0465
	Control	5.0	4.0	4.0	0.0460
Differences between groups	p_2_	ns	0.0107	0.0390	
Weekend days					
Boys	Experimental	4.0	4.0	4.0	ns
	Control	4.0	4.0	4.0	ns
Differences between groups	p_2_	ns	ns	ns	
Girls	Experimental	5.0	4.0	4.0	ns
	Control	4.0	4.0	4.0	ns
Differences between groups	p_2_	ns	ns	ns	

ns—not statistically significant, p_1_ = Friedman’s ANOVA, and p_2_ = Mann–Whitney *U*.
